# The effect of infertility counseling interventions on marital and sexual satisfaction of infertile couples: A systematic review and meta-analysis

**DOI:** 10.18502/ijrm.v20i10.12264

**Published:** 2022-11-02

**Authors:** Somayeh Alirezaei, Ali Taghipour, Robab Latifnejad Roudsari

**Affiliations:** ^1^Department of Midwifery, School of Nursing and Midwifery, Mashhad University of Medical Sciences, Mashhad, Iran.; ^2^Social Determinants of Health Research Center, Mashhad University of Medical Sciences, Mashhad, Iran.; ^3^Nursing and Midwifery Care Research Center, Mashhad University of Medical Sciences, Mashhad, Iran.

**Keywords:** Infertility, Psychosocial intervention, Counseling, Sexual satisfaction, Systematic review, Meta-analysis, Couples.

## Abstract

**Background:**

Psychological consequences of infertility could have a negative effect on marital and sexual satisfaction. Numerous medical associations have strongly recommended psychological interventions, including counseling, to help infertile couples.

**Objective:**

This study reviewed the effectiveness of counseling interventions on marital and sexual satisfaction in infertile couples.

**Materials and Methods:**

This systematic review and meta-analysis was conducted according to the Preferred Reporting Items for Systematic Reviews and Meta-Analyses checklist Databases including PubMed, Web of Science, Psych Info, Cochran Library, Scopus, and Embase were searched for relevant articles published up to March 2020. All randomized clinical trials assessing the impact of psychological interventions on marital and sexual satisfaction in infertile couples were included in the review. The outcome measures were marital and sexual satisfaction, and the pooled estimate of the effects was calculated using a random-effects model. The risk of bias was measured using the Cochrane risk of bias tool, and the summary measures were reported as 95% confidence interval and percentage of heterogeneity.

**Results:**

Out of the 309 studies found through the search, 13 randomized clinical trials including 230 infertile women and 512 infertile couples were systematically reviewed and included in the meta-analysis. It was found that counseling interventions improve marital and sexual satisfaction.

**Conclusion:**

As counseling and psychological interventions increase the marital and sexual satisfaction of infertile couples, those are highly recommended for the psychological management of infertile couples.

## 1. Introduction

Infertility is defined as the inability to achieve pregnancy after 12 months of unprotected intercourse. There are currently 48.5 million infertile couples, of which 19.2 million have primary infertility and 29.3 million have secondary infertility. There are several reasons for infertility, such as male factor, ovulation disorders, tubular problems, endometriosis, sexual disorders, cervical factor, and unexplained causes (1).

Besides, negative physiological and psychological consequences following the diagnosis and treatment of infertility can also have a detrimental effect on marital relationships (2). In most cases, infertility destroys the voluntary and pleasurable aspects of sexual function by setting it as only a means of “having children" (3). This psychological and social burden reduces life satisfaction and increases marital problems (4). Thus, counseling and psychological interventions are needed by couples to combat stress resulting from infertility (5).

There are 5 methods of individual counseling in infertile people: psychodynamic psychotherapy, cognitive-behavioral treatment, strategic psychotherapy, crisis intervention, and grief counseling (6). Strategies used in infertility counseling include the following: marital preparation and promotion programs (7), emotional focus therapy (8), accepting feelings, seeking support, relaxation exercises, talking to the partner, increasing information on infertility and sexual function, sex therapy, and pleasant sexual activity (9).

In a systematic review focused on the effect of counseling interventions on pregnancy rate in infertile patients, it found that although counseling interventions could be beneficial, its effectiveness on sexual function and couple's satisfaction is questionable. In addition, it should be considered that previous meta-analyses had several limitations such as lack of focus on sexual and marital counseling, the high level of heterogeneity, and a number of noncontrolled trials (10).

The efficacy of infertility counseling on marital and sexual satisfaction in infertile couples has not been reviewed. Therefore, this systematic review and meta-analysis aimed to assess the effectiveness of infertility counseling on sexual and marital relationships in infertile couples.

## 2. Materials and Methods

### Literature and search strategy

This systematic review and meta-analysis was conducted based on the preferred reporting items for systematic reviews and meta-analyses checklist (11) (Table I). Researchers searched multiple databases, including PubMed, Web of Science, Psych Info, Cochrane Library, Scopus, and Embase, for English- and Persian-language articles published up to March 2020. The search was performed to find article titles, abstracts, and keywords using words indicating the 3 main topics of “infertility", “counseling", and “marital satisfaction" and “sexual satisfaction”. The keywords included 1) “Infertility", “Childlessness", “In Vitro Fertilization", “Intra Cytoplasmic Sperm Injection" and “Fertility Treatment/Problems", and “Assisted Reproductive Technology" 2) “Psychological/Psychosocial Intervention", “Support", “Counseling", “Psycho-Education", and “Sex therapy" 3) “Marital Satisfaction", “Sexual Function", “Sexual Disorders", and “Sexual Satisfaction". Relevant studies were searched manually to identify further trials missed by the electronic search. 2 authors studied all articles, and disagreements were resolved through consensus with a third reviewer.

**Table 1:** Strategy for systematic searches of the published literature in the database


**#1**	Search “Infertility” OR “Childlessness" OR “IVF* “OR" ICSI* OR “Fertility Treatment/Problems" OR “ART*"	
**#2**	“Psychological/Psychosocial Intervention" OR “Support" OR “Counseling" OR “Psycho-Education" OR “Therapy"	
**#3**	“Marital Satisfaction"	
**#4**	#1 AND #2 AND #3	
**#5**	Identification	Records identified through database searching = 309
	Records removed due to duplication = 29
	Records screened = 280
**#6**	Screening	Records excluded based on title and abstract screening = 250
			Full-text articles assessed for eligibility = 30
		**#7**	Eligibility	Full-text articles excluded = 17
**#8**	Included			Studies included in the systematic review and meta-analysis = 13
*IVF: In vitro fertilization, ICSI: Intra cytoplasmic sperm injection, ART: Assisted reproductive technology		

### Inclusion criteria

The PICO (Patient, Intervention, Comparison, and Outcome) framework was used to identify components of clinical evidence. Experimental and quasi-experimental studies with the following characteristics, which their full text was available, were included in this systematic review and meta-analysis:

P) The target population was infertile patients, either infertile women or infertile couples.

I) The intervention included one of the different approaches of counseling as individual, couple, or group counseling to improve marital and sexual satisfaction.

C) The comparison group was infertile patients who received only routine care.

O) The outcome measures included marital and sexual satisfaction.

### Data extraction 

The titles and abstracts of retrieved articles were reviewed for selecting eligible articles. Next, the selected papers were thoroughly studied to verify they met the inclusion criteria; those that did not were excluded. The remaining articles then thoroughly studied to verify they met the inclusion criteria. Data were extracted separately by 2 researchers, based on a pre-prepared checklist that included the article title, authors, year of publication, journal name, location, number of samples, sampling method, inclusion and exclusion criteria, type of questionnaire to assess the marital and sexual satisfaction of couples, counseling intervention used for infertile couples, reliability and validity of the questionnaire, and the main results.

### Quality assessment and risk of bias 

The Joanna Briggs Institute checklist was used with 2 different versions of experimental and quasi-experimental studies to assess the quality of the articles. The experimental studies evaluation checklist had 13 items. The answer to each item was either “Yes”, “No”, “Unspecified”, or “Not stated”. Score 1 was assigned to each “Yes” while zero for others. The total score of the checklist was 0-13. Based on the obtained scores, the studies were divided into 2 general categories: 1) high-quality studies (score ≥ 6), 2) low-quality studies ( < 6). The evaluation checklist of quasi-experimental studies had 9 items. The total score of the checklist was 0-9, and based on the obtained scores, the studies were divided into 2 general categories: 1) high-quality studies ( ≥ 5) and 2) low-quality studies ( < 5).

The modified Cochrane Collaboration tool was used to assess the risk of bias for randomized controlled trials. Bias was assessed as a result for individual elements from 6 areas of bias: selection bias, performance bias, detection bias, attrition bias, reporting bias, and other bias (12).

### Data analysis and description of findings

All effect size was calculated for marital and sexual satisfaction using the mean difference, weighted with inverse variance, and investigated using the stochastic effects model. Heterogeneity was assessed using Q and I^2^ statistics. This test aimed to check whether the differences in study results were real or random (13). I^2^ is the degree of variance change for the calculated effect size, which results from heterogeneity in studies (14). I^2^ rates include 0%, 25%, 50%, and 75%, indicating no, low, medium, and high heterogeneity, respectively. P ≥ 0.05 was considered significant for heterogeneity assessment. The possibility of publication bias during meta-analysis was investigated using the Funnel plot. Funnel plots are plots of the effect estimates of each study against their measure of precision [standard error] (15).

A meta-analysis was performed based on the obtained results. The calculations were performed using the Comprehensive Meta-Analysis (Version 2; Biostat, Englewood, NJ) software package.

## 3. Results

### Study selection and characteristics

**Figure 1:**
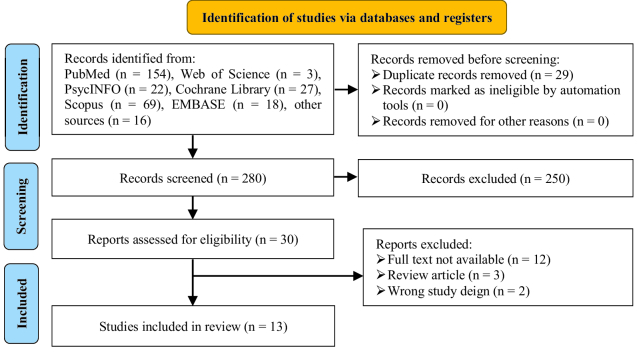
PRISMA 2020 flowchart for selection of articles.

A total of 309 articles were found in the initial search - 154 from PubMed, 3 from Web of Science, 22 from Psych Info, 27 from Cochrane Library, 69 from Scopus, and 18 from Embase. After reviewing the titles and abstracts, 250 articles were excluded from the study. Of the remaining 30 full-text articles, 17 were excluded for not meeting the inclusion criteria, and 13 articles were finally included in the study. Figure 1 presents a flowchart of the selection process.

Moreover, a summary of the article profiles is shown in table II. Thirteen articles including 230 infertile women and 512 infertile couples whose results related to marital and sexual satisfaction were reviewed.

Of the selected 13 articles, 7 were designed and conducted as experimental (randomized clinical trials) (7, 16-21), while 5 were quasi-experimental studies (8, 22, 23). In addition, 11 studies were conducted in Iran, one in Iraq, and one in China. Moreover, 11 studies were published in English while 2 were published in Persian. In all studies, infertile women/couples were treated with ART, but their medical conditions were not fully explained. Of the 13 studies, 8 were conducted on infertile couples and 5 on infertile women. The mean duration of infertility in all studies was 6.5 yr, and the mean age of participants.

Random allocation was performed for all clinical trials. All quasi-experimental studies had a control group. Outcomes including marital and sexual satisfaction were reported as the main result in all studies. While the quality assessment scores of experimental studies were 7 (average quality) to 11 (high quality), it was 7-8 (high) in quasi-experimental studies. In randomized clinical trials studies, none of the studies, except for one (16), met the evaluation criteria No. 4-6, which was related to the blinding method. None of the articles met criterion No. 6 regarding the loss of sample during the study period in quasi-experimental studies. Most studies reviewed the results of the intervention immediately after 3 months. The intervention strategy used was one of the counseling approaches. The duration of each intervention varied from 1-6 hr. The number of counseling sessions was 2-10 sessions per wk. There was no reference to the type of care in the control group in 5 studies. In 8 other studies, it stated that the control group had another caring process as the intervention group. All studies showed no statistically significant difference between demographic and clinical characteristics between intervention and control groups. All studies clearly stated the inclusion criteria for the study.

A review of the studies revealed that they used different counseling methods. One study used mindfulness-based or mindfulness as a psychological intervention. Three studies used the couple's relationship enrichment model (7, 24). Two studies used the emotional focus therapy model (8, 23). Collaborative infertility counseling with problem-focused orientation was used in one study (18). Three studies used cognitive-behavioral theory in counseling (22, 25, 26). One research used reality therapy based on choice theory in their counseling sessions (19). Another study used therapy with active communication skills (16). Finally, one study employed sexual therapy (17).

Different tools used in articles measured marital and sexual satisfaction. The ENRICH Marital Satisfaction Scale was used in 5 studies (7, 22, 25). This questionnaire is used to assess the strengths and weaknesses of marital relationships. It contains 47 questions on a 5-point Likert scale. A score of < 30 equals severe dissatisfaction, 30-40 dissatisfaction, 40-60 relative satisfaction, 60-70 high satisfaction, while > 70 indicates a very high level of marital satisfaction (27). Three studies used a sexual satisfaction questionnaire (16, 17, 26) which included 11 questions that measured the degree of satisfaction with the number of times of intercourse, number of orgasms, level of sexual interest, and attraction during sexual intercourse. It also measures a person's experience of stress during intercourse. A higher score indicates higher sexual satisfaction (17). 4 studies used the index of marital satisfaction to assess marital satisfaction (16, 18, 23, 24). This questionnaire included 25 items with a 5-point Likert scale. The minimum score was 25, and the maximum score was 75, and higher scores indicate higher marital satisfaction (18). One study used Linda Berg's sexual satisfaction questionnaire to assess sexual satisfaction, including 19 items (7). Scores of < 30 indicate low sexual satisfaction, 32-47 show average satisfaction, 48-65 indicate good satisfaction, and > 66 show high sexual satisfaction (28). Besides, 3 studies used the dyadic adjustment scale (1976) to assess marital satisfaction among couples (8, 23), which is a 32-item tool with a minimum score of zero and a maximum score of 160. On this scale, scores < 40 indicate low satisfaction, 40-80 show average satisfaction, 80-120 indicate good satisfaction, and 120-160 show excellent satisfaction with the marital relationship.

**Table 2:** Characteristics of studies included in the systematic review


**Authors (yr)/(Ref) **	**Participants**	**Intervention**	**Tool**	**Results**
		Linda Berg's sexual satisfaction questionnaire	
**Masoumi ** ***et al.*** (2017) (7) Iran ${}^{*}$	50 infertile couples	Couples' relationship enrichment model	Enrich marital satisfaction scale	Marital strengthening training increases marital intercourse and sexual satisfaction in infertile couples
			**Najafi ** ***et al.*** ** (2015)** (8) Iran ${}^{**}$		30 infertile couples	Emotionally focused therapy	Spanier's dayadic adjustment scale	Emotionally focused couples therapy increases intercourse and marital satisfaction in infertile couples and reduces marital and sexual stress
		Index of marital satisfaction	
**Vizheh ** ***et al.*** (2013) (16) Iran ${}^{*}$	100 infertile couples	Communicative skills	Sexual satisfaction questionnaire	Infertility counseling increases marital and sexual satisfaction in infertile couples
			**Pakgouhar ** ***et al.*** (2008) (17) Iran ${}^{*}$		32 infertile couples	Sex counseling	Sexual satisfaction questionnaire	Counseling increases the sexual satisfaction of infertile women
**Latifnejad Roudsari** ***et al.*** ** (2017) (18)** Iran ${}^{*}$	60 infertile women	Collaborative infertility counseling	Index of marital satisfaction	Collaborative infertility counseling increases marital satisfaction in women undergoing infertility treatment
**Ebadi ** ***et al.*** ** (2020)** (19) Iran ${}^{*}$	40 infertile women	Reality therapy based on choice theory	Enrich marital satisfaction scale	Reality therapy based on choice theory could improve marital satisfaction in infertile subjects
**Miri ** ***et al.*** ** (2016)** Iran ${}^{**}$ (20)	30 infertile couples	Relationship enrichment program	Spanier dyadic adjustment scale	Relationship enrichment training is effective on intercourse and sexual and marital satisfaction
**Solati ** ***et al.*** ** (2016)** (22) Iran ${}^{**}$	40 infertile women	Cognitive-behavioral therapy	Enrich marital satisfaction scale	Cognitive-behavioral stress management therapy reduces sexual stress and increases sexual and marital satisfaction in infertile women
**Soleimani ** ***et al.*** (2015) (23) Iran ${}^{**}$	30 infertile couple	Emotionally focused therapy	Spanier dyadic adjustment scale Index of Marital Satisfaction	Emotionally focused couples therapy has a significant effect on sexual satisfaction in infertile couples
**Abedi Shargh ** ***et al.*** (2016) (29) Iran ${}^{*}$	60 infertile women	Mindfulness-based cognitive group therapy	Enrich marital satisfaction scale	Educational and counseling services for infertile women increase their marital satisfaction and reduce sexual problems
**Loke ** ***et al.*** ** (2018)** (24) China ${}^{**}$	100 infertile couple	Partnership and coping enhancement program	Marital satisfaction scale	Enhancement program is feasible and acceptable for improving marital satisfaction of infertile couples
**Hussein Gardi** (2014) (25) Iraq ${}^{*}$	140 infertile couples	Cognitive-behavioral therapy and supportive psychotherapy	Enrich marital satisfaction scale	Psychiatric intervention plays an important role in increasing the satisfaction rate of infertile couples
		Spanier dyadic adjustment scale	
**Ashrafian ** ***et al.*** (2020) (26) Iran**	30 infertile women	Positive cognitive behavioral therapy	Sexual satisfaction scale	Positive cognitive behavior therapy can be used to improve sexual satisfaction and marital adjustment of infertile women
			*Randomized clinical trial design, **Semi-experimental design				

### Risk of bias

**Figure 2:**
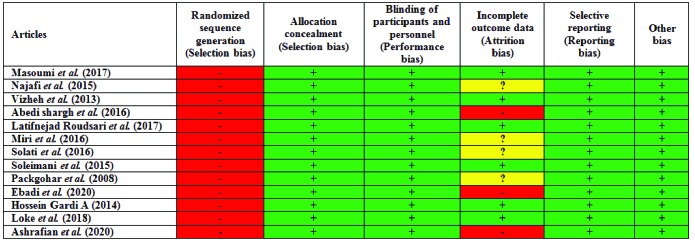
Risk of bias for each study. The green color indicates low risk of bias, yellow indicates unclear risk of bias, and red indicates high risk of bias.

**Figure 3:**
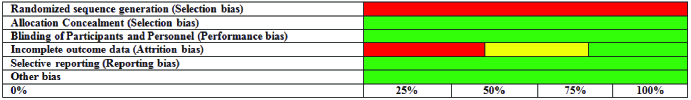
Risk of bias across studies. The green color indicates low risk of bias, yellow indicates unclear risk of bias, and red indicates high risk of bias.

The risk of bias is shown in figures 2 and 3. All studies assessed (100%) were classified as presenting a high risk of bias concerning the random selection of participants. However, regarding random allocation, blinding, implementation, and publication bias, all studies (100%) were classified as presenting a low risk of bias. Concerning attrition bias, 20% of studies were classified as studies presenting a high risk of bias, 35% as an ambiguous risk of bias, and 45% as a low risk of bias. All 13 studies reported the number of participants at the beginning of the study. However, only one study reiterated their number during the follow-up period and at the end of the study (7). Therefore, it was impossible to investigate the sample loss in the intervention and control groups. In addition, there was no report concerning the final number of subjects included in the analysis or the way to deal with lost data.

All studies were evaluated using the standard Joanna Briggs Institute Scale. An important issue in assessing the quality of articles was blinding the intervention for the participants. Due to the nature of counseling for which there is no possibility of blindness, no objections can be made to the studies in this regard. Only one study met the blinding condition (16). This kind of blindness is also explained. The counselor did not know the content of the marriage and sex questionnaires, and the person analyzing the data was not aware of the random assignment of groups.

### Effect of counseling interventions

**Figure 4:**
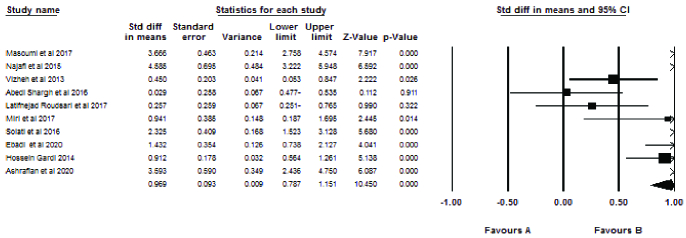
Network meta-analysis for marital satisfaction.

**Figure 5:**
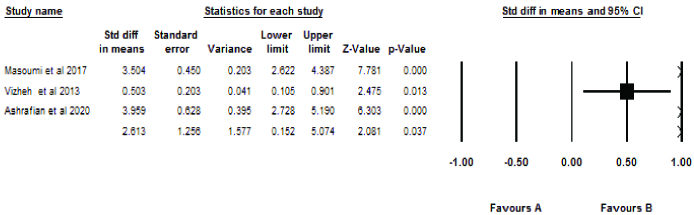
Network meta-analysis for sexual satisfaction.

Figures 4 and 5 show the results of the meta-analysis. The analysis was performed based on the effect size using mean and standard deviation (SD). Since the reported results of 2 studies (25, 30) were not expressed based on mean and SD, they were not included in the meta-analysis.

Ten studies examined marital satisfaction. Their combination showed a significant difference in effect size (ES = 1.74) between the intervention and the control groups. Thus, infertility counseling had a positive effect on marital satisfaction.

Three studies examined sexual satisfaction. Their combination showed a significant difference in the effect size (p = 0.14) between the intervention and the control groups. Therefore, psychological counseling had a positive effect on sexual satisfaction.

Significant Q statistics (p < 0.001) and I^2^ = 93.93 showed heterogeneity between studies. Among the reasons for heterogeneity were the small number of studies, as well as their small sample size.

The effect size of marital satisfaction was significant for both experimental (clinical trial) (mean in diff = 0.71) and quasi-experimental studies (3.436). For sexual satisfaction, the statistical results in experimental studies were 0.80 and 0.75 for quasi-experimental studies.

## 4. Discussion

The results of the meta-analysis showed that infertile people who receive some psychological intervention experience approximately 2.5 times higher marital satisfaction and 1.5 times higher sexual satisfaction than those who don't. The results of 10 studies showed a strong relationship between them, even with evidence of publication bias. The estimated accuracy of the effect size was with a possible mean difference of 0.37-2.42.

The findings of this study are in line with the results of a previous study which evaluated the effectiveness of psychological interventions on infertile patients (6). Moreover, the results of another systematic review and meta-analysis, which examined the effectiveness of psychosocial and psycho-cognitive interventions on infertile men and women, showed that these interventions could be effective for infertile couples, reduce their marital distress, and improve their marital relationships (30). In this study, psychological interventions included counseling, education, relaxation techniques, psychotherapy interventions, problem-solving skills, and information about infertility and marital and sexual issues.

The devastating effect of infertility on a couple's relationship affects all aspects of their lives, and one of the significant effects is marital and sexual relationships (31, 32). Some researchers consider counseling necessary to reduce infertility-related stress and, consequently, to moderate couples' behavior and maintain satisfaction in their relationships (33). Other studies have also reported the supportive impact of infertility counseling on various aspects of infertile couples' life (34, 35).

A 2017 systematic review and meta-analysis identified sexual dissatisfaction as one of the most important sexual disorders in infertile couples (36). According to one study in 2014, a specialized medical and psychological team should be trained to provide strategies for infertile couples to deal with these issues (37). Therefore, to draw definitive conclusions in this study, the effectiveness and sexual and marital satisfaction of infertile patients was investigated immediately after counseling and psychological interventions. It should be considered that any psychological disorder and sexual and marital dissatisfaction would, ultimately, have a negative effect on the effectiveness of treatment and pregnancy in these couples (38).

In this regard, another reason for increasing marital and sexual satisfaction in infertile patients after counseling interventions may be due to the effect of these interventions on other aspects of infertility treatment, such as treatment success (39). Because the overall success of ART depends on the satisfaction level of infertile patients, the lower the couple's dissatisfaction, the higher the success of the treatment. Therefore, the couples that have an adequate marriage and sexual activity under the influence of counseling and consider it free from dissatisfaction are more prepared to accept a variety of treatments and subsequently are more likely to succeed (40). Also, in studies regarding the needs of infertile couples, many participants have highlighted their need for counseling services, psychological support, and strategies for stress management, especially after the treatment failure (41-43).

Among the strengths of the current systematic review and meta-analysis was a comprehensive review based on standard guidelines to find the most appropriate results. A variety of counseling and intervention methods was used in the studies. Besides, a detailed methodology was used to evaluate the articles' quality and effect size.

### Limitations

One of the limitations of this study was that the setting of most studies were the same, all of them were conducted in Iran. Another limitation was the lack of access to the full text of articles from other settings. Moreover, the results obtained regarding heterogeneity between studies acknowledge that the reason may be the small number of articles on the one hand and the small volume of sample on the other. The studies also varied in the follow-up period from an immediate follow-up to 3 months after the intervention. The present study could not provide information on whether the causes or different duration of infertility led to the effect of counseling on the overall outcome. The results of meta-analysis can only answer the question concerning the effect of counseling on marital and sexual satisfaction in infertile patients.

Future research can investigate the impact of different types of psychological interventions in terms of goals (individual, couple, or group interventions), approaches (psychotherapy, counseling, and training), and severity (number and duration) of clinical trial interventions. Future research should also be based on gender differences in counseling infertile people or determining the best counseling method for infertile patients. Studies should accurately consider the causes of infertility, the type of ARTs, and the treatment steps that participants should follow while receiving counseling methods.

Moreover, studies should be conducted in other countries, both developed and developing countries, to make the results generalizable. Counseling for infertile patients should be performed at all infertility diagnosis and treatment stages. It should be emphasized in the field of marital and sexual relationships so that it leads to improvement of the marital and sexual relations of infertile couples. The findings of this study emphasize the supportive role of counseling for infertile couples. Counseling interventions increase the marital and sexual satisfaction of infertile couples.

## 5. Conclusion 

The results of this systematic review and meta-analysis indicate that counseling and psychological interventions increase infertile couple's marital and sexual satisfaction. Therefore, it is recommended that counselors provide counseling interventions to increase the level of infertile couples' marital and sexual satisfaction.

##  Conflict of Interest

The authors declare no conflict of interest and are solely responsible for the writing and content of the article.
